# Studies of Beryllium Chromite and Other Beryllia Compounds With R_2_O_3_ Oxides

**DOI:** 10.6028/jres.064A.008

**Published:** 1960-02-01

**Authors:** C. E. Weir, A. Van Valkenburg

## Abstract

Reactions between BeO and R_2_O_3_ oxides at high temperatures were studied. Compound formation was observed between BeO and the following oxides: B_2_O_3_, Al_2_O_3_, Ga_2_O_3_, Y_2_O_3_, La_2_O_3_, and Cr_2_O_3_. No reaction was observed with Sc_2_O_3_, ln_2_O_3_, and Fe_2_O_3_. Detailed studies were made of BeO·Cr_2_O_3_ which is isostructural with BeO·Al_2_O_3_. BeO·Cr_2_O_3_ is a semiconductor. Optical and X-ray data are given for all reaction products.

## 1. Introduction

In hydrothermal experiments involving the system BeO+Al_2_O_3_+SiO_2_ with small amounts of added Cr_2_O_3_ at 850° C, it was found that X-ray patterns of reaction products persistently showed evidence for an unidentified material. It was found that this material was a compound between BeO and Cr_2_O_3_. The compound has been reported earlier to be BeO·Cr_2_O_3_ by Lang, Roth, and Fillmore [1]^1^ who studied the system BeO-Cr_2_O_3_-ZrO_2_. They found that it was orthorhombic, isostructural with BeO·Al_2_O_3_, with unit call parameters *a*=10.0 A, *b*=5.8 A, and *c*=4.5 A. The present report describes the preparation and some of the properties of BeO·Cr_2_O_3_ as well as preliminary studies on the reactions between BeO and the R_2_O_3_ oxides in group III of the periodic system.

## 2. Experimental Materials and Methods

All reagents used were oxides of reagent grade except Ga_2_O_3_, ln_2_O_3_, and Y_2_O_3_. The former two were made by solution of chemically pure metal in nitric acid followed by thermal decomposition of the nitrate. The available specimen of T_2_O_3_ was of commercial origin with a purity of 99.7 percent.

Hydrothermal reactions were carried out in the well-known cold-seal apparatus [2], using platinum containers. Solid state reactions were made through repeated heating and grinding of oxides. Materials were heated to temperatures as high as 1,550° C in platinum, while BeO containers were used between 1,550° C and 2,100° C. Temperatures to 1,550° C were obtained with platinum wire furnaces and higher temperatures with a carbon resistance furnace using a He atmosphere. Early results indicated the desirability of a rapid screening process. This was accomplished by using a small d-c carbon arc as a furnace. Component oxides were mixed and treated with a sufficient quantity of 10 percent aqueous starch solution to form a paste. The paste was extruded from glass tubing to form small rods ⅛ in. in diameter, which were dried and melted in the arc using the tubing as a handle. All oxides studied were melted rapidly in the arc and a single small fused bead was sufficient to permit X-ray and microscopic analysis. The possible reduction and volatilization of the oxides precludes knowledge of the purity or composition of the arc-fused product, but rapid search for reactions is quite simple with this method.

## 3. Results

### 3.1. BeO·Cr_2_O_3_

This material formed hydro thermally at temperatures as low as 800° C in the presence of water. In this temperature range, however, the reaction rate is very low and crystals are very small.

In the solid state, slow reaction was observed at temperatures as low as 1,400° C. Microscopic comparisons of the surface with the body of specimens showed that prolonged heating of oxide mixtures at 1,450° C produces some surface loss of Cr_2_O_3_. The loss accelerates as the temperature is raised so that only BeO crystals remain on the surface after 4 hr at 1,750° C. Below about 1,600° C the rate of loss is believed to be sufficiently low that the bulk composition is essentially unaffected in normal heating periods. That the Cr_2_O_3_ vapor was at least partially due to vapor pressure of the compound was shown by the fact that BeO·Cr_2_O_3_ sintered in BeO at 1,750° C also showed much surface loss of Cr_2_O_3_. The competitive processes of reaction of the oxides and vaporization of Cr_2_O_3_ from the oxide mixture and the compound indicate that there is an optimum temperature for formation of BeO·Cr_2_O_3_. These experiments indicate that this temperature is probably between 1,300° C and 1,600° C. As a result of vaporization of Cr_2_O_3_ large single crystals would not be expected in open systems; however, crystals as large as 0.05 mm are readily formed under these conditions. The compound forms readily in the arc crystallizing from the liquid phase. Experiments in the arc also showed that the compound BeO·Cr_2_O_3_ was a semiconductor as conductivity of specimens became so high at elevated temperatures that resistance heating could be effected.

Microscopic examination showed that BeO·Cr_2_O_3_ forms well-terminated, transparent, deep green crystals having a reddish pleochroism. The crystals are biaxial negative with 2V≈45° and indices, *α*=2.143, *β* not measured, γ=2.230. Pseudohexagonal, highly twinned crystals similar to those of BeO·Al_2_O_3_ are obtained on reaction via the liquid phase.

Poly crystalline material appears black and opaque. It has a hardness on the Moh’s scale of about 9 by the scratch test. The melting point was not measured but it was found that a eutectic between BeO and BeO·Cr_2_O_3_ melts below approximately 2,050° C. The measured specific gravity is 4.42 and the principal X-ray powder lines are given in table 1 with the indices and unit cell parameters. X-ray studies conducted on mixtures rich in BeO or Cr_2_O_3_ showed no appreciable solid solution of the parent oxides in BeO·Cr_2_O_3_.

**Figure 1:**
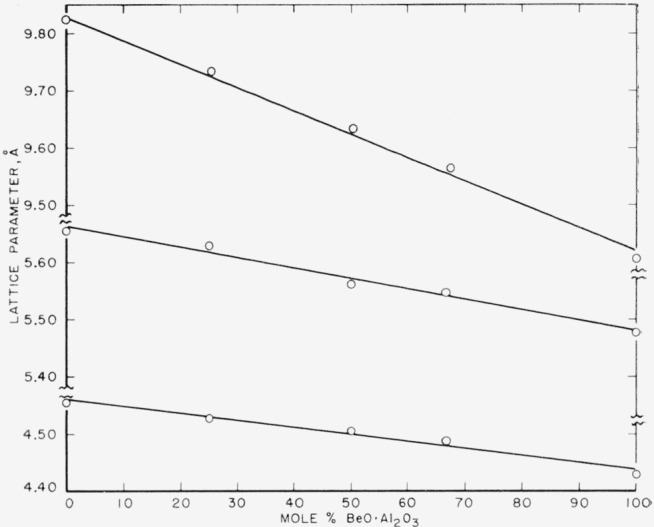
Variation of unit cell parameters in the solid solution series BeO·Al_2_O_3_−BeO·Cr_2_O_3_.

X-ray powder patterns show BeO·Al_2_O_3_ and BeO·Cr_2_O_3_ are isostructural. Several solid solutions were prepared from the component oxides and the variation of the unit cell parameters with composition is shown in figure 1. Although some curvature is possible in the variation of "a" with composition, the deviations from linearity are probably within the experimental error of determining the cell parameters. In addition the compositions denoted by the points are determined from the known quantities of oxides added and may be subject to some error arising from loss of Cr_2_O_3_ on firing. This error is believed to be small. From these data it appears that a complete series of solid solutions exists between BeO·Al_2_O_3_ and BeO·Cr_2_O_3_. This conclusion differs from that of Gjessing, Larrson, and Major [3] who found only partial substitution of Cr_2_O_3_ for Al_2_O_3_ in the BeO·Al_2_O_3_ structure.

**Figure 2:**
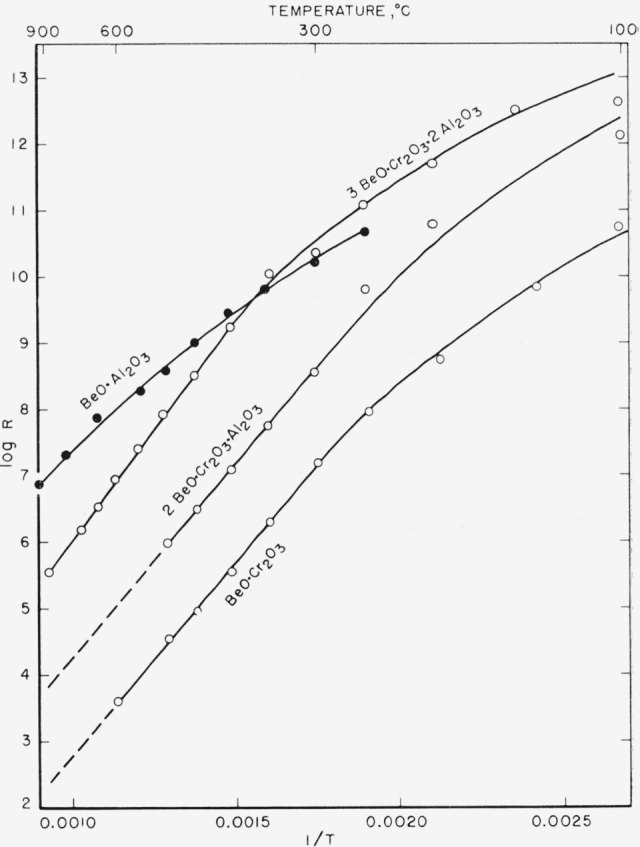
Temperature variation of resistance of BeO·Al_2_O_3_, BeO·Cr_2_O_3_ and solid solutions.

In view of the electrical conductivity of BeO·Cr_2_O_3_ at elevated temperatures, it was of interest to determine the temperature-resistance characteristics. These measurements were made on polycrystalline sintered bars using platinum electrodes. Specimens were measure in a temperature-controlled furnace using a commercial megohm bridge which applied 500 v across the specimen. Since single crystals were not used, and the bars possessed pores, the resistivities could not be determined, the resulting data being valid only for showing the temperature dependence of resistance. The resistance was measured at temperature intervals of approximately 100° C and the data are shown for BeO·Cr_2_O_3_ as well as BeO·Al_2_O_3_ and two solid solutions in figure 2. The data are plotted in the usual manner using 1/*T* and log R. From the figure it will be observed that data for specimens containing Cr_2_O_3_ are not inconsistent with the interpretation that they are intrinsic semiconductors, above a hout 300° C. If this interpretation is correct the average energy gap is calculated to be 2.4 ev and appears to be independent of the Al_2_O_3_/Cr_2_O_3_ Ratio.

The magnetic characteristics of BeO·Cr_2_O_3_ are being studied by W. E. Henry of the Naval Research Laboratory. Preliminary results show the material to be antiferromagnetic.

### 3.2. BeO-R_2_O_3_ Reactions

#### a. BeO−B_2_O_3_

A crystalline product is formed by heating BeO and crystalline B_2_O_3_ at 800° C. The principal X-ray lines for the material are given in table 2. Crystals are biaxial with 2V≈90° and indices *α*=1.62, *β* not measured, *γ*=1.574. The material is not isostructural wit h BeO·Al_2_O_3_. From the X-ray pattern the product can he identified with the compound 3BeO·B_2_O_3_ reported by Menzel and Sliwinski [4] and by Mazelev [5].

#### b. BeO-Al_2_O_3_

In addition to BeO·Al_2_O_3_, the product BeO·3Al_2_O_3_ is readily formed by are fusion of the 1:3 oxide mixture. This material is identified with the X-ray powder pattern given originally by Foster and Royal [6] and extended by Budnikov et al. [7].

#### c. BeO-Sc_2_O_3_

Mixtures of BeO and Sc_2_O_3_ can be fused with difficulty in the arc. However, despite numerous attempts no evidence for reaction was observed. X-ray powder patterns of fused beads showed only BeO and Sc_2_O_3_ lines.

#### d. BeO-Ga_2_O_3_

Arc fusion studies showed reaction between BeO and Ga_2_O_3_, hut X-ray patterns showed the product was not isostructural with BeO-Al_2_O_3_. Gjessing, Larrson, and Major [3] have previously reported a compound, BeO-2Ga_2_O_3_, which is not isostructural with BeOAl_2_O_3_. Solid state reactions indicated that the material was readily formed at 1,350° from a mixture of oxides. Although complete reaction was not obtained in short periods at this temperature as shown by X-ray lines of unreacted BeO or Ga_2_O_3_ the product is believed to be identical with the BeO-2Ga_2_O_3_ reported earlier [3]. Microscopic examination showed the material to be uniaxial positive with indices 1.747 and 1.774. The X-ray powder pattern lines given in table 2 were obtained by deleting the known lines of BeO from the pattern. This pattern has been indexed in the hexagonal system with *a*=7.78 A and *c*=2.98 A, with the indices given in table 2.

#### e. BeO-Y_2_O_3_

Arc fusion studies showed reaction between BeO and Y_2_O_3_. All attempts to produce a similar material by solid state reactions at temperatures as high as 1,600° C were unsuccessful. Therefore the composition as well as the purity of the material are not known. Microscopic examination showed biaxial crystals with 2V≈90 and indices, *α*=1.84, *β* not measured, *γ*=1.85. The X-ray powder pattern lines are listed in table 2 but it should be noted that extraneous lines from impurities introduced by the arc may be present. This material is obviously not isostructural with BeO-Al_2_O_3_ and does not appear to have been reported previously.

#### f. BeO-In_2_O_3_

Repeated efforts at high and low temperatures, in open and closed systems, both with and without water present failed to give any evidence for reaction. Ensslin and Valentiner [8] reported BeO-In_2_O_3_ as having the same lattice constant as ln_2_O_3_. Gjessing, Larrson, and Major [3] found no reaction. On the basis of the present work it is concluded that reaction between the oxides does not occur to 1,600° C.

#### g. BeO-La_2_O_3_

These oxides react readily in the arc. Solid state reactions may be carried out at 1,300° C and indicate that the product is probably 2BeO La_2_O_3_. Microscopic examination shows biaxial crystals with 2V ≈ 90° having indices, *a*= 1.995, *β* not measured, 7=2.047. X-ray powder pattern lines are given in table 2.

#### h. BeO-Cr_2_O_3_

In addition to the compound BeO·Cr_2_O_3_ the question of a 1:3 compound analogous to BeO·3Al_2_O_3_ was investigated. Evidence was found that only the 1:1 compound was formed, X-ray patterns showing lines for BeO·Cr_2_O_3_ and Cr_2_O_3_ only.

#### i. BeO-Fe_2_O_3_

Several experiments were performed to prepare the Beo-Fe_2_O_3_ analog of BeO-Al_2_O_3_ starting from the oxides. None were successful. In most instances Fe_3_O_4_ was formed. The question of the existence of BeO·Fe_2_O_3_ from previous work is not clear [9, 3]. However, from the present experiments it appears that such a compound does not form from the oxides at temperatures up to the liquidus.

## 4. Summary

High temperature reactions of BeO with the R_2_O_3_ oxides have been studied. The compound BeO·Cr_2_O_3_ has been studied in some detail. It is isostructural with BeO·Al_2_O_3_ and a continuous series of solid solutions forms between these compounds. The unit cell parameters in the solid solutions show nearly ideal behavior. BeO·Cr_2_O_3_ is a semiconductor.

Compound formation was verified between BeO and the following oxides: B_2_O_3_, Ga_2_O_3_, Y_2_O_3_, and La_2_O_3_. Powder X-ray diffraction patterns are given for all compounds and that for BeO-2Ga_2_O_3_ is indexed in the hexagonal system. No reaction was observed between BeO and Sc_2_O_3_, ln_2_O_3_, and Fe_2_O_3_.

## Figures and Tables

**Table 1:** *X-ray powder pattern for* BeO·Cr_2_O_3_ Cu K*_α_*, λ=1.5405

BeO·Cr_2_O_3_^1^		
*d*_obs._	*hkl*	*d*_calc._

*A*		*A*
4.135	101	4.131
3.337	111	3.337
2.653	301	2.654
2.452	220	2.451
2.402	311	2.403
2.335	121	2.335
2.157	401	2.157
1.936	321	1.936
1.715	$\{\begin{array}{c} 421\\ 511 \end{array}$	1.715
		1.716
1.668	222	1.669
1.539	331	1.538
1.519	003	1.519
1.4159	040	1.4155
1.4140	620	1.4141
1.3765	303	1.3771

1 Unit cell parameters *a*=9.792 A, *b*=5.663 A, *c*=4.555 A; calculated density 4.654 g/cm^3^ at 25° C.

**Table 2:** *X-ray powder patterns for* BeO·R_2_O_3_ *compounds* Cu Kα, λ = 1.5405

3BeO·B_2_O_3_		BeO·2Ga_2_O_3_				nBeO·Y_2_O_3_		nBeO·La_2_O_3_	

*d, A*	*I*	*d, A*	*hkl*^1^	*I*	*d*_calc_.	*d, A*	*I*	*d, A*	*I*
7.302	vw	6.717	100	m	6.716	7.190	w	3.708	vs
6.067	w	3.875	110	m	3.877	5.196	w	3.718	m
5.921	w	3.359	200	s	3.358	4.607	vw	3.673	m
3.967	m	2.728	101	s	2.723	4.469	s	3.296	m
3.872	vs	2.539	210	vs	2.538	3.146	m	3.063	m
3.826	m	2.230	202	s	2.228	2.922	vs	3.008	s
3.635	m	1.938	220	w	1.939	2.838	m	2.681	m
3.188	s	1.934	211	w	1.932	2.786	m	2.628	m
2.901	s	1.863	310	w	1.863	2.765	w	2.620	m
2.863	w	1.791	301	m	1.790	2.600	w	2.612	s
2.718	w	1.626	221	w	1.625	2.479	w	2.494	m
2.480	m	1.579	311	w	1.579	2.412	w	2.465	w
2.446	w	1.541	320	w	1.541	2.213	w	2.224	w
2.365	w	1.489	002	w	1.495	2.193	w	2.167	m
2.204	w	1.465	410	w	1.466	2.060	w	2.127	m
2.168	w	1.369	321	m	1.369	1.979	m	2.055	w
2.061	w	1.316	111	w	1.316	1.975	w	2.030	w
1.932	w	1.286	212	w	1.286	1.945	w	1.980	m
1.901	w	1.269	420	vw	1.269	1.888	w	1.965	w
1.751	W	1.207	510	vw	1.206	1.767	m	1.883	m
1.737	w	1.183	222	vw	1.182	1.705	w	1.838	w
1.656	w	1.169	421	vw	1.168	1.682	w	1.820	w
1.430	w	……..	……..	……..	……..	1.631	w	1.785	m
1.275	w	……..	……..	……..	……..	1.460	w	1.703	m
						1.455	w	1.740	m
						1.289	w	1.700	w
								1.698	w
								1.678	w
								1.652	w
								1.647	w
								1.638	w
								1.627	w
								1.610	w
								1.608	w
								1.542	w
								1.515	w
								1.483	w
								1.446	m
								1.209	w
								1.201	m

1 Indexed on the basis of a hexagonal unit cell with parameters *α* = 7.78 A and *c*=2.98A.

## Footnotes

1 Figures in brackets indicate the literature references at the end of this paper.
